# Peritoneal Lymphomatosis Mimicking Advanced Ovarian Malignancy: A Diagnostic Challenge in Diffuse Large B-Cell Lymphoma

**DOI:** 10.7759/cureus.107136

**Published:** 2026-04-16

**Authors:** Daniel Kyeremateng, Lora Umelue, Maisie Black, Chipo Ndlovu

**Affiliations:** 1 Gastroenterology, New Cross Hospital, Wolverhampton, GBR; 2 Elderly Care Medicine, New Cross Hospital, Wolverhampton, GBR; 3 Stroke Medicine, New Cross Hospital, Wolverhampton, GBR; 4 Acute Medicine, New Cross Hospital, Wolverhampton, GBR

**Keywords:** ascites, ca125, diffuse large b-cell lymphoma, lymphadenopathy, non-hodgkin lymphoma, omental caking, ovarian malignancy mimic, peritoneal carcinomatosis, peritoneal lymphomatosis

## Abstract

Diffuse large B-cell lymphoma (DLBCL) may rarely present as peritoneal lymphomatosis, closely mimicking peritoneal carcinomatosis on imaging and creating significant diagnostic uncertainty. We report the case of an 82-year-old woman presenting with progressive dyspnoea, fatigue, and systemic symptoms following treatment for presumed community-acquired pneumonia. Contrast-enhanced computed tomography demonstrated ascites, diffuse peritoneal thickening, omental involvement, and widespread lymphadenopathy, initially raising suspicion for advanced ovarian malignancy, supported by an elevated CA125 level. Excisional cervical lymph node biopsy confirmed DLBCL with peritoneal involvement. A biopsy was performed shortly after imaging, and there was no significant delay in establishing the diagnosis. Given the patient's frailty, supportive care was pursued. This case highlights the importance of recognising peritoneal lymphomatosis as a key diagnostic mimic of ovarian malignancy and emphasises the role of early tissue diagnosis when imaging findings suggest peritoneal carcinomatosis.

## Introduction

Non-Hodgkin lymphoma (NHL) accounts for the majority of lymphoma diagnoses and may present with a wide range of extranodal patterns [1]. Diffuse large B-cell lymphoma (DLBCL) is the most common aggressive subtype and can occasionally involve sites outside the lymphatic system [1].

Peritoneal lymphomatosis is an uncommon manifestation of lymphoma characterised by tumour infiltration of the peritoneum [2]. Radiologically, this can closely mimic peritoneal carcinomatosis, producing imaging findings, such as ascites, diffuse peritoneal thickening, and omental caking [2,3]. These features are more typically associated with metastatic ovarian or gastrointestinal malignancy and may therefore lead clinicians down a carcinoma-focused diagnostic pathway [2-4].

This diagnostic challenge may be further compounded by elevated CA125, a tumour marker commonly associated with ovarian malignancy, but which may also rise in conditions causing peritoneal irritation or serosal inflammation [5,6].

We report a case of DLBCL presenting with classical radiological features of peritoneal carcinomatosis and markedly elevated CA125, initially raising suspicion for advanced ovarian malignancy. This case highlights an important diagnostic mimic and emphasises the importance of early tissue diagnosis when imaging suggests peritoneal carcinomatosis. Misinterpretation as ovarian malignancy may delay recognition of lymphoma, a potentially treatable disease if diagnosed early.

## Case presentation

An 82-year-old woman, previously independent, presented with five weeks of progressive dyspnoea, fatigue, productive cough, reduced appetite, and bilateral lower limb swelling. She had completed two outpatient antibiotic courses for presumed community-acquired pneumonia without improvement.

Her past medical history included uterovaginal prolapse and haemorrhoids. She was not on regular medication and had no known drug allergies. Prior to this illness, she was living independently, driving, and managing all activities of daily living (baseline Eastern Cooperative Oncology Group (ECOG) performance status 1). On examination, she was tachycardic and tachypnoeic but normotensive, with oxygen saturations of 94% on air. She was alert and oriented. Respiratory examination revealed bilateral crackles with reduced air entry at the lung bases. The abdomen was soft but mildly distended with clinical evidence of ascites. No hepatosplenomegaly was detected. A right cervical lymph node was palpable on further assessment. No significant abnormalities were identified on per rectal or pelvic examination.

Chest radiography demonstrated bilateral pleural effusions. Initial laboratory investigations are summarised in Table 1.

**Table 1:** Laboratory results on admission LDH = lactate dehydrogenase; CA125 = cancer antigen 125 Reference ranges based on local laboratory standards.

Parameter	Result	Reference Range	Units
Haemoglobin	102	115-160	g/L
White blood cell count	12.8	4.0-11.0	×10⁹/L
C-reactive protein	145	<5	mg/L
Albumin	24	35-50	g/L
Lactate dehydrogenase (LDH)	620	135-225	U/L
Cancer antigen 125 (CA125)	337	<35	U/mL

**Figure 1:**
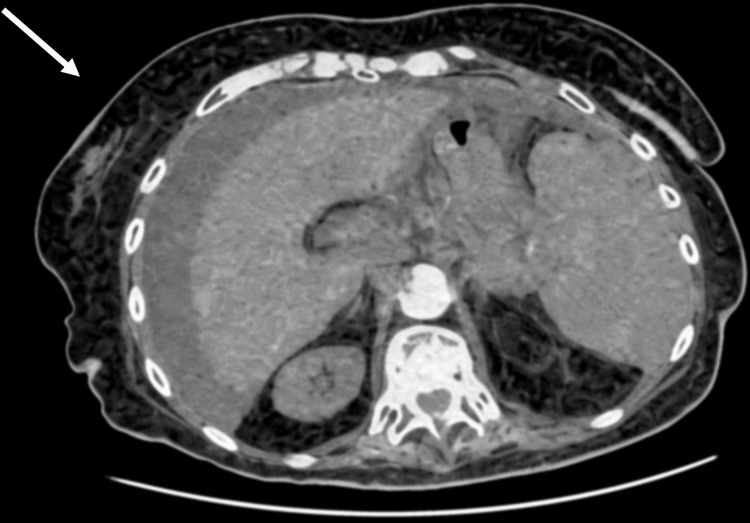
Axial contrast-enhanced CT image demonstrating omental involvement Axial contrast-enhanced CT image demonstrating increased soft tissue density within the anterior abdomen (arrow), suggestive of omental involvement.

Given persistent symptoms and concern for an underlying malignant process, contrast-enhanced computed tomography (CT) of the thorax, abdomen, and pelvis was performed. This revealed diffuse peritoneal thickening with omental involvement, moderate ascites, and extensive mesenteric and para-aortic lymphadenopathy. Additional mediastinal and cervical nodal involvement was also identified (Figure 1).

Although ovarian malignancy was initially suspected, further gynaecological investigations, such as transvaginal ultrasound, were not pursued, as the presence of extensive lymphadenopathy above and below the diaphragm was atypical for a primary ovarian malignancy and prompted prioritisation of tissue diagnosis. The combination of ascites, omental caking, and elevated CA125 raised suspicion for peritoneal carcinomatosis secondary to ovarian malignancy [5-7]. However, the presence of widespread lymphadenopathy above and below the diaphragm suggested lymphoma as an important alternative diagnosis [2-5].

**Figure 2:**
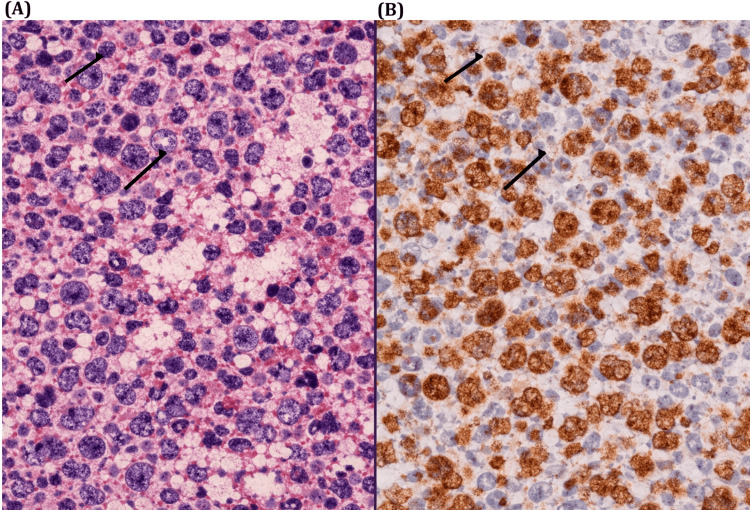
Histopathology and CD20 immunostaining of the cervical lymph node (A) H&E (×400) showing heterogeneous lymphoid infiltrate with large atypical cells (arrows). (B) CD20 (×400) showing patchy membranous positivity in tumour cells (arrows), confirming B-cell lineage.

Histopathological examination of the excised cervical lymph node demonstrated partial effacement of nodal architecture by a heterogeneous population of lymphoid cells, including large atypical forms with vesicular chromatin and prominent nucleoli, admixed with smaller lymphocytes (Figure 2A). Immunohistochemical staining showed patchy membranous positivity for CD20 in the large atypical cells, confirming B-cell lineage (Figure 2B).

Given her advanced age, declining functional status, and overall frailty, she was not considered a suitable candidate for intensive chemotherapy. Following multidisciplinary discussion, she declined disease-modifying treatment in favour of comfort-focused care. Therapeutic ascitic drainage was performed for symptomatic relief, and she was subsequently transferred to hospice care. PET-CT, although useful in staging lymphoma, was not performed as histological diagnosis had already been established, and further staging would not have altered management.

## Discussion

Peritoneal lymphomatosis is a rare extranodal manifestation of NHL but represents a clinically important mimic of peritoneal carcinomatosis [2,3]. Ascites, diffuse peritoneal thickening, and omental caking are classically interpreted as metastatic spread from ovarian or gastrointestinal malignancy, and these imaging appearances often trigger a carcinoma-focused diagnostic pathway [2-4,8,9]. Radiological overlap between malignant peritoneal diseases is well recognised, and distinguishing primary peritoneal pathology from secondary involvement can be particularly challenging on imaging alone [8,9].

Other differential diagnoses for diffuse peritoneal disease include tuberculous peritonitis, primary peritoneal mesothelioma, and metastatic gastrointestinal malignancy. These conditions may produce overlapping radiological features, such as ascites, peritoneal thickening, and omental involvement. However, the presence of extensive lymphadenopathy, particularly involving nodal stations above the diaphragm, favours lymphoma [2-5,8,9].

This case demonstrates how lymphoma can reproduce this radiological pattern almost exactly, creating substantial diagnostic uncertainty at presentation. The elevated CA125 further reinforced suspicion for advanced ovarian malignancy despite its lack of specificity. Similar cases of peritoneal lymphomatosis mimicking ovarian malignancy have been reported in the literature [3,5]. Although frequently regarded as a gynaecological tumour marker, CA125 may rise in any condition causing peritoneal irritation or serosal inflammation, including lymphomatous infiltration [5-7].

Radiologically, peritoneal lymphomatosis may be differentiated from peritoneal carcinomatosis by the presence of bulky lymphadenopathy, particularly involving nodal stations above the diaphragm [2-5,10]. Prior imaging studies have highlighted that extensive nodal disease, especially when distributed both above and below the diaphragm, is a key feature favouring lymphoma over primary peritoneal malignancy [10]. This distribution is atypical for primary ovarian malignancy and should prompt consideration of lymphoma, even in the presence of ascites and omental involvement. Key distinguishing imaging features are summarised in Table 2.

**Table 2:** Radiological features distinguishing peritoneal carcinomatosis from peritoneal lymphomatosis Adapted from Cabral et al. and Kim et al. [2,10]

Feature	Peritoneal Carcinomatosis	Peritoneal Lymphomatosis
Primary cause	Metastatic ovarian or gastrointestinal malignancy	Non-Hodgkin lymphoma
Ascites	Common	Common
Omental caking	Common	May occur
Peritoneal thickening	Common	Common
Bulky lymphadenopathy	Uncommon	Characteristic finding
Nodal disease above diaphragm	Rare	Frequently present
CA125 elevation	Common	Possible due to peritoneal irritation

Misclassification as ovarian malignancy may lead to referral down gynaecological cancer pathways, delaying recognition of lymphoma, a potentially treatable condition if identified early. Previous radiological-pathological correlation studies have emphasised the importance of integrating imaging findings with clinical and laboratory features to avoid premature diagnostic closure in cases of diffuse peritoneal disease [8,9].

The key diagnostic clue in this case was the presence of extensive, bulky lymphadenopathy above and below the diaphragm, extending into the cervical region. Such widespread nodal disease is atypical for primary ovarian carcinoma and should prompt consideration of lymphoma, even when the peritoneal pattern appears malignant [2-5,10].

Ultimately, definitive diagnosis depended on early tissue biopsy. Clinicians should maintain lymphoma as a differential diagnosis in patients presenting with diffuse peritoneal disease, particularly when imaging findings are accompanied by widespread lymphadenopathy. Even in the context of palliative management, tissue diagnosis remained essential to confirm the underlying pathology and avoid misclassification as ovarian malignancy, which would have led to a fundamentally different management approach.

This distinction is clinically important, as management differs significantly. DLBCL is an aggressive but potentially curable malignancy, typically treated with immunochemotherapy such as R-CHOP (rituximab, cyclophosphamide, hydroxydaunorubicin, oncovin, and prednisone) [1]. In contrast, advanced ovarian malignancy is generally managed with cytoreductive surgery and platinum-based chemotherapy. Accurate diagnosis is therefore essential to ensure appropriate treatment pathways [5-7].

This case highlights the importance of maintaining a broad differential diagnosis when evaluating patients with diffuse peritoneal disease. While peritoneal carcinomatosis is commonly associated with advanced ovarian or gastrointestinal malignancy, the presence of extensive lymphadenopathy - particularly involving nodal stations above the diaphragm - should prompt consideration of lymphoma. Early tissue biopsy remains essential, as radiological appearances and tumour markers alone may be misleading. Recognising this diagnostic pitfall may help clinicians avoid premature diagnostic closure and ensure timely investigation of potentially treatable haematological malignancies.

## Conclusions

DLBCL may rarely present with extensive peritoneal involvement, closely mimicking advanced ovarian malignancy. Lymphoma should remain a differential diagnosis in patients with ascites, omental caking, and widespread lymphadenopathy, even when tumour markers suggest a gynaecological primary. Early tissue biopsy is essential to avoid diagnostic misclassification and guide appropriate management.
